# Transcriptome and Proteome-Based Network Analysis Reveals a Model of Gene Activation in Wheat Resistance to Stripe Rust

**DOI:** 10.3390/ijms20051106

**Published:** 2019-03-04

**Authors:** Hong Zhang, Ying Fu, Huan Guo, Lu Zhang, Changyou Wang, Weining Song, Zhaogui Yan, Yajuan Wang, Wanquan Ji

**Affiliations:** 1 State Key Laboratory of Crop Stress Biology for Arid Areas, College of Agronomy, Northwest A&F University, Shaanxi 712100, China; zhangh1129@nwafu.edu.cn (H.Z.); fuying2008@126.com (Y.F.); guohuan2018@163.com (H.G.); zhanglu162049@163.com (L.Z.); chywang2004@126.com (C.W.); sweining2002@yahoo.com (W.S.); wangyj7604@163.com (Y.W.); 2 College of Horticulture and Forestry Sciences, Huazhong Agricultural University, Wuhan 430070, China; gyan@mail.hzau.edu.cn; 3 Shaanxi Research Station of Crop Gene Resource & Germplasm Enhancement, Ministry of Agriculture, Shaanxi 712100, China

**Keywords:** wheat, stripe rust, iTRAQ, WGCNA, transcriptome-proteome associated analysis, splicing regulator

## Abstract

Stripe rust, caused by the pathogen *Puccinia striiformis* f. sp. *tritici* (*Pst*), is an important fungal foliar disease of wheat (*Triticum aestivum*). To study the mechanism underlying the defense of wheat to *Pst*, we used the next-generation sequencing and isobaric tags for relative and absolute quantification (iTRAQ) technologies to generate transcriptomic and proteomic profiles of seedling leaves at different stages under conditions of pathogen stress. By conducting comparative proteomic analysis using iTRAQ, we identified 2050, 2190, and 2258 differentially accumulated protein species at 24, 48, and 72 h post-inoculation (hpi). Using pairwise comparisons and weighted gene co-expression network analysis (WGCNA) of the transcriptome, we identified a stress stage-specific module enriching in transcription regulator genes. The homologs of several regulators, including splicing and transcription factors, were similarly identified as hub genes operating in the *Pst*-induced response network. Moreover, the Hsp70 protein were predicted as a key point in protein–protein interaction (PPI) networks from STRING database. Taking the genetics resistance gene locus into consideration, we identified 32 induced proteins in chromosome 1BS as potential candidates involved in *Pst* resistance. This study indicated that the transcriptional regulation model plays an important role in activating resistance-related genes in wheat responding to *Pst* stress.

## 1. Introduction

Stripe rust, caused by the pathogen *Puccinia striiformis* f. sp. *tritici* (*Pst*), is an important fungal foliar disease of wheat (*Triticum aestivum*) found in many areas of the world. Although different techniques have been used to control the disease, the use of stripe rust-resistant varieties is the safest, most cost-effective and environmentally-friendly approach. Selection of disease-resistant varieties using traditional methodology is laborious; however, many stripe rust-resistance genes/fragments have been transferred into wheat using traditional intergeneric and interspecific cross strategies [1]. The greatest challenge facing wheat crop breeders is the ability of pathogens to overcome specific resistance due to the narrowing genetic diversity of the germplasm and rapid variation in the stripe rust pathogen [2,3] Dissecting the disease-resistance mechanism in search of the resistant (R) genes in wheat has become a focus of research. Over the years, numerous comprehensive analyses of stress-induced gene expression in model plants with small genomes have led to the identification of many genes associated with responses to pathogenic stress. To be infective, the pathogen must breach the plant cell wall, and often, the host plasma membrane. In response to pathogenic attack, plants may deploy basal and R gene-mediated defense mechanisms involving the pathogen-associated molecular pattern-triggered immunity (PTI) and/or effector-triggered immunity (ETI) systems [4]. The use of classical genetic techniques to isolate R genes in wheat has met with limited success due to the hexaploid nature of the wheat genome, which is larger and more complex than that of model plants. Wheat-*Pst* interactions have been investigated using RNA sequencing (RNA-Seq) [5,6], GeneChip [7], cDNA-AFLP analysis, and degradome sequencing [8]. Thousands of functional genes and non-coding genes involved in the responses of wheat to pathogen invasion have been identified in these studies. It is no doubt that more R genes will be detected using new technology, such as Renseq [9]. However, classical genetics studies have shown that the resistant trait is a monogenic control phenotype, making it almost impossible to identify the key R gene from transcription expression profiles. Furthermore, the transcriptional changes detected are not necessarily reflected in the expression of the corresponding determinative proteins due to post-transcriptional, translational, and/or post-translational regulatory mechanisms [10] and the complexity of alternative splicing [11]. As a result, the mechanism underlying wheat resistance activation in response to *Pst* remains to be fully elucidated.

Proteomics technologies are key tools used to study complex biological processes at the protein level. Examples of such technologies include isobaric tags for relative and absolute quantification (iTRAQ), which has been employed successfully in a plant proteomics studies [12,13]. In a recent study of changes in the protein expression profiles of wheat resistant to the pathogen that causes powdery mildew (*Blumeria graminis* f. sp. *tritici*; *Bgt*), we detected upregulation of several stress- and defense-related proteins in response to infection [14]. This significantly narrowed the number of potential defense-related proteins, which was an important step in the identification of proteins that are critically involved in resistance to *Bgt* infection. Weighted gene correlation network analysis (WGCNA) is used to delineate both weighted and un-weighted correlation networks using big data. This method can be used to generate testable hypotheses for validating independent datasets, such as the modules associated with receptacle development [15], macrophage activation and flavonoid biosynthesis [12]. The wheat line N9134 has maintained a high level of resistance to both stripe rust and powdery mildew because of two resistance genes; one located in the short arm of chromosome 1B and the other in the long arm of chromosome 5B, respectively [16]. Transcriptome comparisons in the winter wheat introgression N9134 resistant line revealed activation of various genes involved in antagonizing infection by stripe rust and powdery mildew pathogens [5]. In the present study, the iTRAQ-based quantitative proteomic technique was used to study changes in the protein expression profile of *Pst*-inoculated N9134 seedling leaves. Furthermore, we narrowed the field of potentially critical resistance genes by creating networks based on merging data generated by RNA-Seq following weighted gene co-expression network analysis (WGCNA) with the results of transcriptome-proteome-associated analysis. The main objective was to identify a spectrum model of wheat resistance activation in response to stripe rust pathogen infection by comparing changes in transcriptomic and proteomic profiles after inoculation with *Pst*. A complete overview of the organization of the transcriptome was obtained by WGCNA. This method is used to elucidate both the higher-order relationships between genes based on their co-expression as well as groups of biologically-related genes, known as “modules”, comprising the core functional units of the transcriptional network. Within each module, the most highly connected or central genes, are referred to as “hubs”. Taking the classical genetics resistance gene locus into consideration, we used gene expression patterns and the co-expression relationships to construct the regulative module network of wheat responding to stripe rust infection with an edge weight higher than 0.1. Finally, we delineated a module associated with core transcription processes, including spliceosome and transcript regulator function, in the early stages of the response of the N9134 wheat resistant line to *Pst*-inoculation.

## 2. Results

### 2.1. Global Analysis of iTRAQ Data

**Figure 1:**
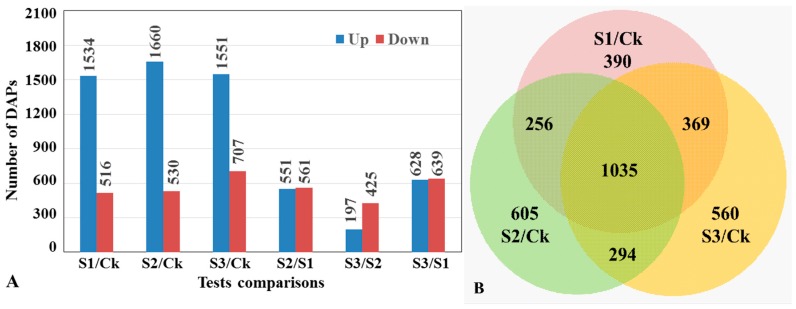
Number of differentially accumulated proteins (DAPs) in wheat infected by stripe rust. (**A**). Bar chart. The blue and red bars represent up- and down-regulated DAPs, respectively. The numbers of test comparisons are shown above each bar. S1, S2, and S3 represents the samples of *Pst*-inoculated at 24, 48, and 72 hpi, while Ck means the mock-inoculated sample. (**B**). Venn diagram. The number of specific and overlapped DAPs between timepoints were given.

A total of 373,661 mass spectra were generated from iTRAQ analysis of *Pst*-inoculated N9134 leaves. The raw mass data files were deposited in the PeptideAtlas database: PASS00999. After excluding low-scoring spectra, 46,174 unique spectra that matched to specific peptides were obtained. Of these, 12,265 matched with 20,898 unique peptides and 8314 protein species were quantified. These peptides and proteins were further filtered using the following criteria simultaneously: (1) >1.2-fold change in protein expression comparing with control; (2) *t*-test *p* < 0.05; (3) differential accumulated expression detected in at least two out of three biological replicates. Compared with the 0 hpi control, 2050, 2190, and 2258 protein species were identified as significant differentially accumulated proteins (DAPs) in the *Pst*-infected samples at 24, 48 and 72 hpi, respectively (Figure 1). 

**Figure 2:**
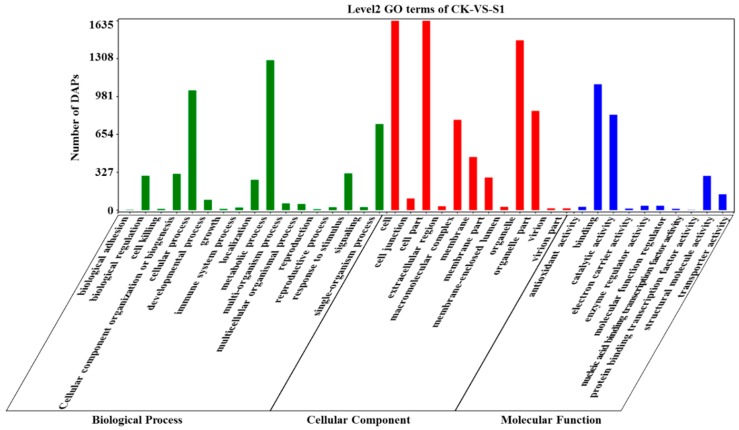
Gene ontology (GO) classifications of the differentially accumulated proteins (DAPs) identified in isobaric tags for relative and absolute quantification (iTRAQ) of *Pst*-infected wheat leaves at 24 hpi. The level2 GO terms are shown below each bar, while ontology classes biological process, Cellular component and molecular function are represented by green, red and blue bars, respectively. The number of DAPs was shown as the ordinate.

In gene ontology (GO) enrichment analysis, the main molecular functions of the DAPs identified in *Pst*-inoculated N9134 leaves were classified as binding, catalytic activity, transporter activity, and structural molecule activity (Figure 2). Of which, RNA binding, protein binding, nucleic acid binding, inorganic cation transmembrane transporter activity and acid anhydrides hydrolase activity were especially significantly enriched with *p*-value < 0.0001. This result was consistent with findings reported under *Bgt* stress [14]. However, the ratio of DAPs classified in the electron carrier and enzyme regulator activity molecular function categories in the present study was much lower than the number associated with *Bgt* stress. In GO analysis of the DAPs identified in *Pst*-inoculated N9134 leaves at 24 hpi, 2,050 DAPs were classified as various cellular components, such as cell part, organelle, macromolecular complex and membrane. Similar results were obtained in GO enrichment analysis of *Pst*-inoculated N9134 leaves at 48 and 72 hpi (Figure S1), with very little change in the abundance of DAPs. 

To identify the biological pathways that were active in *Pst*-inoculated N9134 leaves at 24, 48 and 72 hpi, the DAPs were further investigated by Kyoto Encyclopedia of Genes and Genomes (KEGG) pathways analysis. When the FDR-corrected *p*-value was set at 0.05, 22 significantly enriched KEGG pathways induced by *Pst* stress were identified (Table 1). Significant differential enrichment was found for ribosome, oxidative phosphorylation, plant-pathogen interaction, and glycine, serine, and threonine metabolism pathways at 24, 48, and 72 hpi. In comparison, significant differential enrichment was found for phagosome, circadian rhythm-plant, and flavonoid biosynthesis at 24 and 48 hpi only; for glutathione metabolism, carbon metabolism, basal transcription factors, citrate cycle, glyoxylate and dicarboxylate metabolism, and riboflavin metabolism at 48 and 72 hpi only; and for arginine and proline metabolism, one carbon pool by folate, biosynthesis of amino acids, sulfur metabolism, alanine, aspartate and glutamate metabolism, monobactam biosynthesis, and selenocompound metabolism at 48 hpi only. These results indicated that the activated pathways played primary roles in N9134 wheat responding to *Pst* infection. More importantly, 48 hpi appeared to be the more important time-point for protein expression in the resistance of N9134 to *Pst* infection as nearly all of the significantly enriched pathways were detected at this stage comparing to 24 and 72 hpi.

### 2.2. PPI Network Construction for DAPs Induced by *Pst* Stress

**Figure 3:**
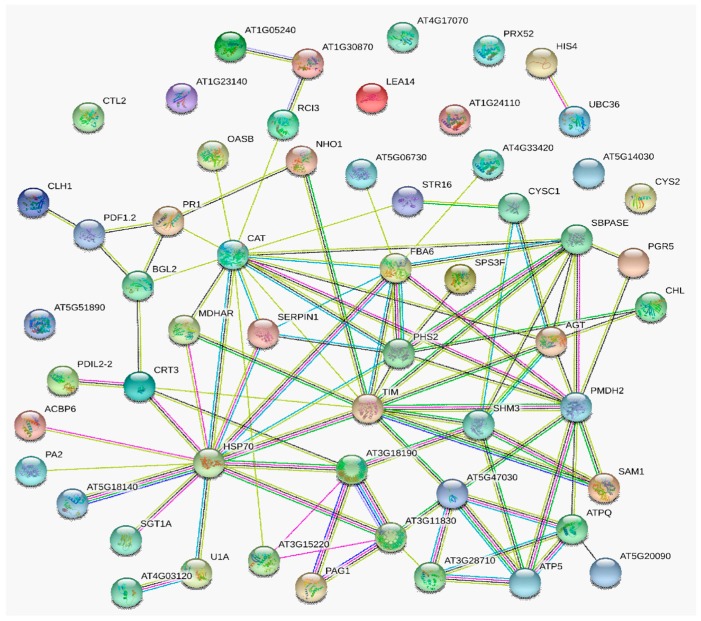
Interaction networks of response to stress related DAPs identified by isobaric tags for relative and absolute quantification (iTRAQ). Protein–protein interaction network constituted by DAPs identified in N9134 responding to *Pst* stress and known protein-species related to defense and response to biotic stimulus in the Database, Experiment, or Text Mining databases. The purples lines represent experimental evidence. The green lines represent gene neighborhood, while the blue lines represent gene co-occurrence database evidence. The yellow lines represent textmining evidence; and the black lines represent the co-expression evidence. U1A, Spliceosomal protein U1A; AT4G03120, C2H2 and C2HC containing protein (Component of the U1 snRNP C); TIM, Triosephosphate isomerase; AT3G11830, TCP-1/cpn60 chaperonin family protein; SHM3, Serine hydroxymethyltransferase 3; SGT1, Suppressor of the G2 allele of skp1; NHO1, Glycerol kinase; PR1, Pathogenesis-related gene 1; MDHAR, Monodehydroascorbate reductase; OASB, Cysteine synthase; AGT, Alanine-glyoxylate aminotransferase; ATPQ, ATP synthase subunit d; PDIL2-2, PDI-like 2-2(protein disulfide isomerase); TIM, Triosephosphate isomerase; CAT, Catalase 2; AT1G30870, Peroxidase 7; AT1G05240, Peroxidase 1/2; AT5G51890, Peroxidase 66; CTL2, Chitinase-like protein 2.

To further investigate the interactions among *Pst* stress-induced protein-species, DAPs predictively related with defense response to stress/stimulus were integrated with information from STRING database to construct a PPI network (Table S1). Three interaction networks were predicted from 56 nodes proteins with the enrichment *p*-value < 1.0 × 10^16^ (Figure 3) at the medium confidence parameter level. Hsp70 was a key protein-species in this powerful PPI network and interacted with SGT1, MDHAR, TCP-1/cpn60 chaperonin, PDIL2-2, ATPQ, SHM3, OASB, TIM, CAT, ERDJ3A, U1A, and U1C. From Figure 3, we can see the interaction between NHO1 with MDHAR and PR1. This means that Hsp70 may have some interactive relationship with NHO, although the directly interactions was not observed. Additionally, SHM3 and TCP-1/cpn60, acted as protein-folding catalyst activity, were predicted as the second important hub nodes. The other two predicted PPI networks were shown in Figure 3 as well, including ubiquitin-conjugating enzyme E2 and Peroxidase 1/2 interacted with Histone H4 and Peroxidase 7 separately. 

### 2.3. Co-Expression Network Analysis of Wheat Resistance to Stripe Rust

**Figure 4:**
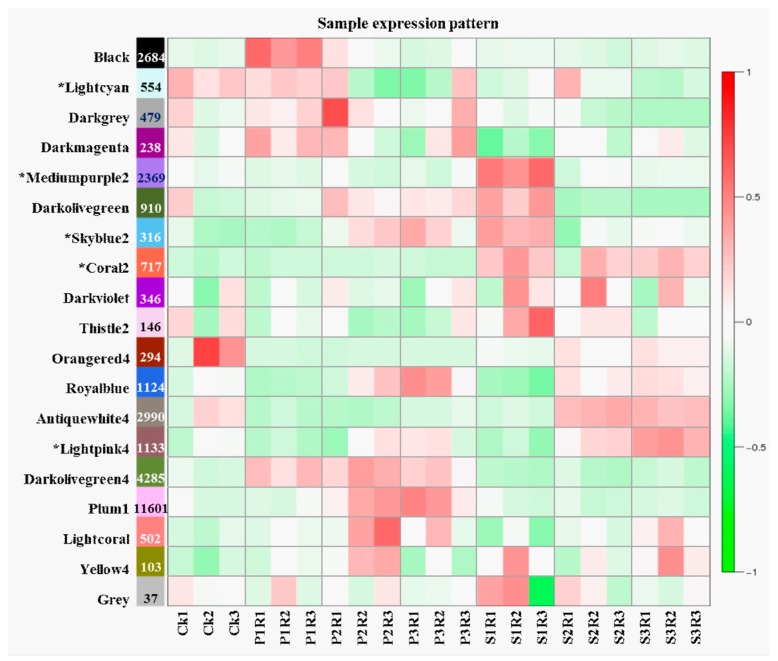
Weighted gene co-expression network analysis of genes in *Pst*- and *Bgt*-infected wheat young leaves tissues. The expressed genes were clustered into 18 modules labeled by different colors (not gray). Each row corresponds to one module. The number of genes in each module is indicated on the left. Each column corresponds to a specific sample. The color of each cell at the row-column intersection indicates the correlation coefficient between the module and the sample. The value of the correlation between a specific module and sample is indicated by the scale bar on the right. The letter P and S in X-axis referred to the *Bgt* and *Pst* stress respectively, while the following number 1, 2, and 3 represent the samples collected at 24, 48, and 72 hpi after inoculation. Similarly, R1, R2, and R3 mean the replications and sample number. The specific modules of wheat responding to *Pst* are marked with an asterisk.

Co-expression network analysis was performed by comparing 21 high-throughput RNA-Seq datasets generated from leaf samples at the four stages under two fungal stress levels with three repeats. For *Pst*-infected leaf samples, the average number of high quality clean reads per library was approximately 35.99 million 101 bp paired-end reads compared to 37.42 million clean reads from the *Bgt*-infected samples (PRJNA243835). Based on bread wheat reference transcripts, a total of 66,727, 60,890, 65,482, and 64,243 genes were assembled for samples obtained at 0, 24, 48, and 72 hpi, respectively. Co-expression networks were also evaluated by WGCNA. In the present study, co-expression networks were constructed on the basis of pairwise correlations between *Pst* stress responsive genes using their common expression trends across all sampled leaves. Modules are defined as clusters of highly interconnected genes; genes within the same cluster have high correlation coefficients, with each tree branch constituting a module and each leaf representing one gene. We identified 79 distinct modules (labeled by different colors), which were merged into 18 main modules with mergeCutHeight of 0.3 (Figure S2). The module eigengene was considered to be representative of the corresponding module’s gene expression profile in wheat responding to *Pst* stress. The module eigengenes from the 18 main modules were each correlated with distinct samples as sample-specific eigengene expression profiles (Figure 4). Four of the 18 co-expression modules: mediumpurple2, skyblue2, coral2 and lightpink4, consisted of genes that were highly expressed in the *Pst* test type (*r* > 0.8, *p* < 10^−3^). In contrast, the lightcyan co-expression modules consisted of genes with down-regulated expression. These five modules each identify (or correlate with) a time-point or specific cluster of genes. For example, as shown in Figure 4, the skyblue2 module identified 316 *Pst*-induced specific genes at 24 hpi and the lightcyan module identified 554 *Pst*-repressed specific genes at 24 and 72 hpi.

Among these modules, mediumpurple2 and lightpink4 comprised the most sample-specific expressed genes. These genes were enriched mainly in photosynthesis-antenna proteins, plant hormone signal transduction, amino acid, and carbon metabolism pathways. These results were in accordance with those of previous studies using common enrichment analysis [5] (Table S2, Figure S3). The genes in coral2 module were enriched in ribosome, porphyrin and chlorophyll metabolism, and limonene and pinene degradation KEGG pathways, while the skyblue2 and lightcyan modules consisted of genes enriched in spliceosome, glutathione metabolism, phagosome, ascorbate and aldarate metabolism, and photosynthesis pathways. This result suggested that these five modules represent the primary gene expression module that characterized the specific response of N9134 to *Pst* race CYR 31 stress.

### 2.4. The *Pst*-Induced Module Transiently Expresses Genes Enriched in Splicing and Transcription Activity

**Figure 5:**
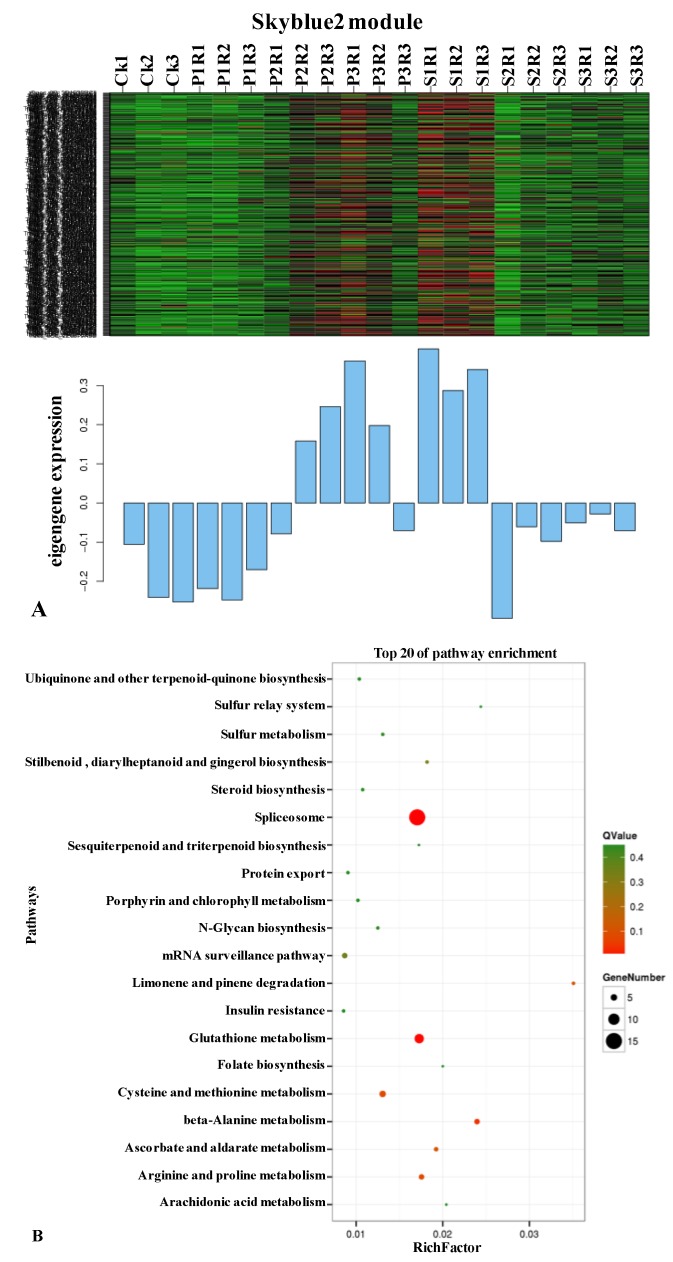

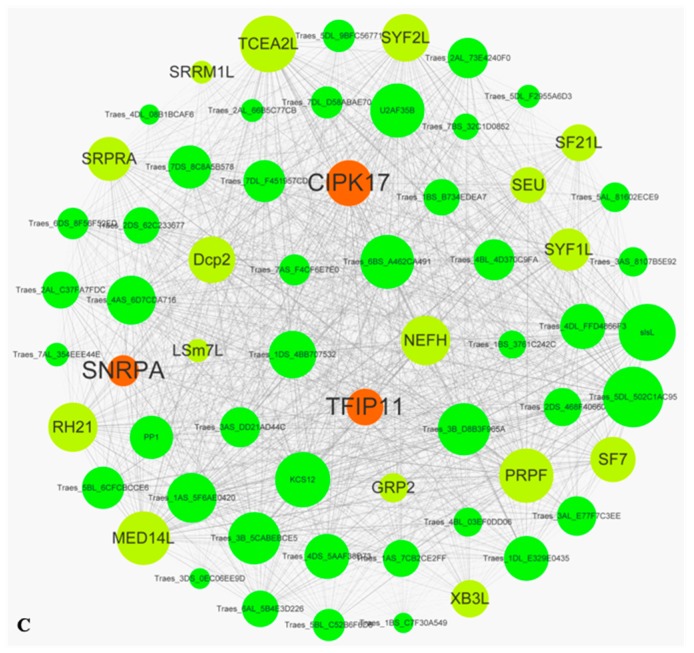
Construction of Pst-induced specific gene modules and networks. (**A**). Heat map and eigengene expression profile for the skyblue2 (24 hpi) module in leaves of *Pst*-inoculated wheat resistance line N9134. The y-axis indicates the value of the module eigengene; the x-axis indicates sample type. The letter P and S in X-axis referred to the *Bgt* and *Pst* stress respectively, while R showed the replications. Heat map shows the relative FPKM of each gene. (**B**). The top 20 KEGG pathway enrichment categories of differentially expressed genes (DEGs) in skyblue2 module. The KEGG pathways are listed on the left, while Q-values and gene numbers are shown on the right. (**C**). The correlation network of the skyblue2 module. Sixty-six genes with edge weight >0.1 are visualized using Cytoscape_v.3.0.0. Twenty-three genes related to spliceosome components or transcription-related regulators are marked with abbreviated gene names, while the others were marked with URGI gene numbers. The diameter of the circles is proportional to connectivity.

Of the 18 main modules, the modules containing the genes specifically induced in the early stages (especially in 24 hpi) of *Pst* infection, mediumpurple2, coral2, and skyblue2, were of particular interest. The mediumpurple2 consisted of 2369 genes. Enriched KEGG pathways responsible for signal transduction and photosynthesis energy pathways were detected in earlier protein enrichment analysis. In the coral2 module, 717 differentially expressed genes (DEGs) functioned in polypeptide translation and compound metabolism. The skyblue2 module contained 316 genes that were not detected in DAP enrichment analysis. The heat map showing the weighted relative FPKM of each gene from the skyblue2 module revealed that many module genes were strongly expressed at 24 hpi with *Pst* (Figure 5). These genes were enriched in spliceosome, glutathione metabolism, beta-alanine metabolism, arginine and proline metabolism, cysteine and methionine metabolism, ascorbate and aldarate metabolism, and limonene and pinene degradation (*p* ˂ 0.01). Similar results were also detected in profile 18 by the earlier k-means clustering method (Figure S4). In profile 18, the gene expression pattern was steeply up-regulated at 24 hpi and then returned to levels similar to those of the control. These results suggested that wheat expresses related genes to influence spliceosome function after signal recognition by changing the transcripts to prevent stripe rust pathogen infection. 

WGCNA was also performed to construct transcription expression gene networks, in which each node represents a responsive gene and the connecting lines (edges) between genes represent co-expression correlations. Hubs consist of the genes that show the most connections in the network. In the skyblue2 module network (Figure 5C), 32 out of the 268 genes (37 from 316 loci due to wheat polyploidy) encoded regulators related to transcription. Details of the highlighted genes in the network are shown in Table S3. Some of the hub genes, including serine/threonine protein kinase, calcium-dependent protein kinase (CDPK) and CBL-interacting protein kinase (CIPK) in signal transduction pathways, RNA-binding Musashi-2-like protein and Ras-related proteins that regulates apoptosis and endocytosis, were defense-related. The spliceosome pathway-related genes were mainly expressed in two patterns as shown in profile 7 and profile 18 with 27 and 20 genes, respectively (Table S4). Most of the genes in profile 7 showed significant down-regulation at 24 hpi comparing with the levels at 0 hpi, while the expression levels were increased at 48 hpi, and remained stable at 72 hpi; this pattern of expression is hereafter referred to as ‘down-up-even’. The expression levels of the genes in profile 18 differed from those in profile 7, with an up-down-even pattern over the 0–72 hpi time period (Figure S4). This result indicated that the spliceosome pathway was dysregulated in wheat variety N9134 at 24 hpi after *Pst* infection and that spliceosome activation may plays an important role in the mechanism underlying gene activation in wheat resistance to stripe rust.

**Figure 6:**
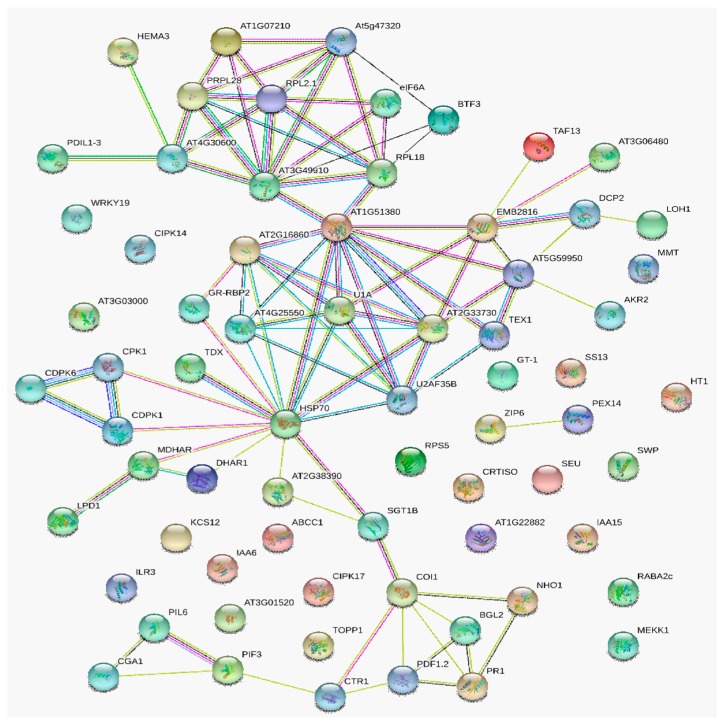
Interaction networks of defense-related DAPs identified by iTRAQ with differential expressed genes in skyblue2 module together. Protein–protein interaction network constituted by DAPs and DEGs identified in N9134 responding to *Pst* stress. The purples lines represent experimental evidence. The green lines represent gene neighborhood, while the blue lines represent gene co-occurrence database evidence. The yellow lines represent textmining evidence; and the black lines represent the co-expression evidence. HSP70, Heat shock protein 70; U1A, Spliceosomal protein U1A; U2AF35B, Splicing factor U2af small subunit B; DCP2, mRNA-decapping enzyme subunit 2; AT2G16860, pre-mRNA-splicing factor SYF2; AT2G33730, DEAD-box ATP-dependent RNA helicase 21; AT1G51380, DEAD-box ATP-dependent RNA helicase 34; PRPL28, 60S ribosomal protein L28-1; AT3G49910, 60S ribosomal protein L26-1; AT4G25550, Cleavage/polyadenylation specificity factor; AT3G03000, Putative calcium-binding protein CML18; GR-RBP2, Glycine-rich RNA-binding protein 2; PIL6, Transcription factor PIF5; RPS5, Resistant to p. syringae 5; CDPK6, Calcium-dependent protein kinase 6; PR1, Pathogenesis-related gene 1.

Considering that the nodes degrees of U1A and U1C were similarly observed in protein network, we re-constructed a PPI network combining the DEGs in skyblue2 transcription module with defense-related DAPs from proteome data (Table S5). The results showed that Spliceosomal protein U1A, pre-mRNA-splicing factor SYF2, Splicing factor U2af small subunit B, Cleavage/polyadenylation specificity factor, Ribosomal protein S19, Cleavage/polyadenylation specificity factor, and DEAD-box ATP-dependent RNA helicase 21 and RNA helicase 34 were detected in the key hub node with HSP70 together from 69 nodes proteins with the enrichment *p*-value < 1.37 × 10^11^ (Figure 6). The resistance protein, defensin, was interacted with BGL2, CTR1, COI1, and pathogenisis-related protein PR1 directly. Moreover, the indirectly interaction between HSP70 with CDPK6 could be predicted via calcium-dependent protein kinase CPK1 or CDPK1 as an intermedium protein. The KEGG pathways enrichment results showed that the PPI network protein significantly enriched in spliceosome, plant-pathogen interaction, mRNA surveillance, plant hormone signal transduction, and MAPK signaling pathway. This means that splicing activity maybe an important trigger of defense gene against pathogen infection, although the directly interactions networks still need to be verified and further analyzed in following investigations.

### 2.5. Screening for Disease-Resistance Priming Genes in N9134 Using Transcriptome-Proteome-Associated Analysis

**Figure 7:**
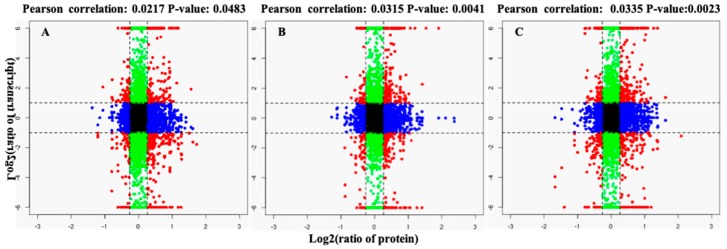
Transcriptome-proteome-associated analysis of gene expression in Pst-infected wheat leaf tissues. The x-axis indicates the level of DAP accumulation detected in the proteomic analysis, while the y-axis indicates the level of DEG expression detected in the transcriptome analysis. The Pearson correlation p-values are indicated above each plot. A, B, and C represent different analysis at 24, 48, and 72 hours after inoculation, respectively. Each plot is divided into nine quadrants (1, 2, 3 at the top; 4, 5, 6 in the middle; and 7, 8, 9 at the bottom, ordered from left to right), which indicate the correction of gene expression at the transcript and proteins levels.

Most R genes encode proteins containing nucleotide binding and leucine-rich repeat (NLR) domains [17]. A typical plant genome contains hundreds of NLR-encoding genes in each chromosome [18], with many forming complex clusters. However, in classical genetics, most R genes are known to be responsible for a monogenic control phenotype in which defense-related downstream genes are activated by priming or core control genes/loci. The enriched modules and up-regulated DAPs were screened for R gene regulators by transcriptome-proteome-associated analysis (Figure 7). The stripe rust R gene is a dominant locus in wheat variety N9134, and located in the short arm of chromosome 1B; therefore, we focused mainly on 1BS annotated candidate genes in the reference genome. In total, 32 candidate genes in chromosome 1BS were up-regulated at the transcript and/or protein level in response to *Pst* stress; details of these genes are shown in Table 2. Half of these genes were related to genetic information processing and regulation, plant hormone signal transduction and oxidative phosphorylation. Several peroxisome pathway-related genes, such as the peroxisomal membrane proteins PEX2, PEX14-like and dehydroascorbate reductase, were also identified. Additionally, some genes, including CBL-interacting protein kinase 17, p23 (a co-chaperone of Hsp90), subtilisin-chymotrypsin inhibitor-2A, serine/threonine protein phosphatase PP1 isozyme 9 and SUN domain-containing protein 3-like, were identified as resistance response-related genes. Among this group of genes, SUN domain-containing protein 3-like and ribosomal protein L28 gene had the highest connectivity with other hub genes (1385 and 975.6, respectively). Interestingly, no candidate genes encoding disease-resistant proteins were identified in the definite genomic interval, although 187 resistance gene analogs were detected in gene transcripts in routine RNA-Seq analyses. Considering the interactions of gene loci in classical genetics, this result indicated that the “commander” gene may have the capacity to deploy the resistance-related gene in response to the pathogen infection.

## 3. Discussion

### 3.1. Splicing Activity May Play an Important Regulatory Role in Plant Responses to Fungi

The *Pst* pathogen is responsible for significant losses in grain production in the wheat-growing areas with moist climates worldwide. Thus, studies and identification of genes responsible for resistance to *Pst* infection are both of economic and scientific significance. In the present study, we performed triplicate iTRAQ proteome surveys in young leaves of the winter wheat resistance line N9134 inoculated with *Pst*. We identified 37 node genes encoding transcription-related regulators in the skyblue WGCNA module at the 24 hpi stage under *Pst* stress. The production of these regulators in wheat suggests that wheat plant transcriptional activity, including pre-mRNA splicing to form new transcripts and changes in transcript expression levels, may undergo rapid readjustment in response to *Pst* infection. Specifically, we found versatile elements of the spliceosome in the specific module induced by *Pst*. The first example is the U1 small nuclear ribonucleoprotein A, which is a component of snRNP protein complexes that interact with cell division cycle 5-like (CDC5L) protein [19]. Second, the splicing factor 3A (SF3A) subunit 3 is a component of the non-snRNP protein SF3A [20], which is essential for activating the U2 snRNP. Third, pre-mRNA processing factors (PRPF), which are associated with proteins of different snRNPs, such as PRPF8 (a component of both U2- and U12-dependent spliceosomes) [21], PRPF3 and PRPF4 (associated with U4 and U6 snRNPs) [22], and PRPF6 (involved in pre-mRNA splicing as a bridging factor between U5 and U4/U6 snRNPs in spliceosome formation) [23,24]. U1 small nuclear ribonucleoprotein A, SF3A, and PRPFs function in certain spliceosomes to perform pre-mRNA splicing that generates multiple transcript variants encoding different isoforms. The THO complex subunit protein 4 is a nuclear protein and functions as a molecular chaperone. Virbasius et al. postulated that THO complex subunit protein 4 is involved in regulating DNA binding, dimerization, and transcriptional activity of basic region-leucine zipper proteins [25]. The THO complex is also known to play a role in transcription-dependent recombination, transcription itself, and transcriptional elongation [26]. THO knockdown affects the processing of the transcript’s 3ʹ-end of transcripts encoding heat shock protein 70 (hsp70) in *Drosophila* [27,28]. The THO/TREX complex appears to play functional roles in gene transcription, as well as processing and nuclear export of mRNAs. Furthermore, some proteins of the THO/TREX complex, such as glycine-rich RNA-binding protein 2 (GRP2), are involved in autoregulation. The AtGRP8 homolog promotes the generation of an alternatively spliced transcript containing a premature termination codon to limit functional AtGRP8 protein production [29]. Taken together, our findings represent a platform for further exploration of the upstream genes in the disease defense system in hexaploid wheat and furthermore, may be important in explaining the recent report of the host–pathogen interaction that induces temporally coordinated waves of gene expression in wheat [6].

### 3.2. Resistance Gene Regulators May Be More Important in Understanding the Pathogen Defense Mechanism in Wheat

In response to the presence of pathogens, plants have evolved innate immune systems to initiate effective defense responses via the PTI and ETI pathways. Accumulation of resistance genes has been reported with notable differences in the mechanisms responsible for *Pst*-resistance in wheat [5,30]. In general, *Pst*-infected host plants and host responses show monogenic control that is typical of classical genetics. Surprisingly, R genes containing classical NLRs in chromosome 1BS were not identified in the wheat resistance line N9134. However, we identified three apparent differences in the mechanisms underlying *Pst* resistance in wheat. First, the expression pattern of a SUN homolog in wheat leaves correlated with the path of *Pst* invasion. The plant inner nuclear membrane (INM) Sad1/UNC-84 (SUN) proteins were first identified in *Arabidopsis* [31], and two types have been identified; the classical C-terminus SUN proteins (Cter-SUN) and the internal SUN domain (mid-SUN) proteins [32]. The AtSUN–SINE2 complexes are involved in defense against pathogens, plant-microbe symbiosis, and physical stress responses [33,34]. In this study, up-regulated SUN proteins showed the highest connectivity (up to 1385) with the other hub genes that responded to *Pst* stress, strongly indicating that SUN proteins play an important role in regulating defense-related genes. Thus, the precise details of the function of SUN proteins in anti-*Pst* responses warrant further investigation. Second, p32, which is an important co-chaperone for the Hsp90 chaperoning pathway [35], was found to be significantly up-regulated at the protein level and showed high connectivity (399.7) in the WGCNA network. Hsp90s play key roles in controlling seedling growth and stripe rust disease resistance in common wheat [36,37], although a similar role for p32 in wheat-*Pst* resistance remains to be determined. The third difference consisted of the implication of serine/threonine protein phosphatase 1 (PP1) and calcineurin B-like (CBL)-interacting protein kinase (CIPK) 17 in protection of wheat seedlings against *Pst* infection. CBLs interact with the conserved NAF domain at the CIPK C-terminus to release the kinase from auto-inhibition [38]. Different CIPKs are recruited competitively by various CBLs to provide versatility in responding to Ca^2+^ signals and the subsequent immune responses, such as hypersensitive cell death [39,40]. Serine/threonine protein phosphatases PP1 and PP2A play key roles in apoptosis, which is a genetically programmed form of cell death [41]. Interestingly, CIPKs interact with PP2Cs by binding the kinase domain of CIPK and the protein phosphatase interacting motif (PPI) in the regulatory domain [42]. Additionally, phytochrome-interacting factor (PIF3), a nuclear helix-loop-helix transcription factor that binds to G-box DNA-sequence elements in light-regulated promoters, was also detected in relation to *Pst* stress, which is accordance with previous reports of light perception in disease defense signaling and induction of pathogenesis-related genes in wheat [43,44,45]. Increased PIF activity has been demonstrated to promote the expression of genes encoding enzymes involved in auxin synthesis [44]. Interestingly, we detected increased expression of auxin-responsive protein IAA15 in the present study. This evidence strongly supports a role for the predicted candidate genes in regulating plant R genes in response to *Pst* infection. 

Several transcriptomes and proteomes have been reported to decipher the mechanism underlying the responses of plants to pathogen stress. Due to limitations in the analytical methods used, previous studies have focused mainly on the downstream genes, including those related to the resistance phenotype and secondary metabolite biosynthesis. By combining the generation of co-expression networks, transcriptome-proteome-associated analysis and classical genetic methodology, we hypothesized the functional roles of the spliceosome complex activity in gene regulation and expression in wheat infected with *Pst* for the first time. This discovery set the foundation for experimental investigation using applicable reverse genetics resources in wheat [46], and will be finally beneficial in further elucidating the defense mechanism deployed in wheat against pathogen invasion. We speculate that there are two possible roles for these R gene regulators. First, they may modulate signals that trigger defense responses in both resistant and susceptible plants. R gene regulators are known to exist in the Chinese spring cultivar that is susceptible to *Pst* CYR 32, but contains the slow rusting resistance gene loci *Lr34*/*Yr18*/*Pm38* [47]. Second, in the early stages of pathogen invasion, these R gene regulators may differentially activate the defense pathway of the plant through different levels of expression. Our study provides further elucidation of the defense mechanisms deployed in wheat in response to *Pst* infection and may have wider implications for studies of other plant R genes.

## 4. Material and Methods

### 4.1. Plant Materials and Pathogen Stress Treatment

The winter wheat introgression line N9134, which shows high resistance to *Pst* races CYR 31 with a single resistant genetic locus on chromosome 1BS [5], was developed at Northwest A&F University (NWAFU). The *Pst* race CYR 31 was a gift from the College of Plant Protection of NWAFU. Ten-day-old seedlings were brush-inoculated with *Pst* CYR 31 urediniospores collected from “Mingxian 169” seedlings infected 20 days previously. The wheat landrace “Huixianhong” was used to monitor inoculation efficiency. Following *Pst*-inoculation N9134 leaf samples (consisting of three biological replicates) were harvested at 24, 48, and 72 h post-inoculation (hpi); mock-inoculated leaves were harvested at 0 hpi. The developmental period of four time points is about from 2.5-leaf to 2.8-leaf stage. All samples were frozen immediately in liquid nitrogen, and were stored at −80 °C. After the monitor varieties Huixianhong were fully sporulating, the samples were used for experiment.

### 4.2. Protein Extraction, iTRAQ Labeling, and LC-ESI-MS/MS Analysis

Total proteins were extracted from leaf tissue samples at the four stages using the optimized extraction procedure described by Fu et al. [14]. Protein samples (100 μg) were digested with Trypsin Gold (Promega, Beijing, China) solution (protein:trypsin, 30:1) at 37 °C overnight. The resultant peptides were then dried by vacuum centrifugation, reconstituted in 0.5 M tetraethyl-ammonium bicarbonate and processed with the 8-plex iTRAQ reagent according to the manufacturer’s protocol. Peptides were labeled with different isobaric tags (Table S6), pooled and re-dried under vacuum. The labeled peptide mixtures were then re-dissolved and purified according to the standard iTRAQ protocol. The pool of labeled samples was subjected to high performance liquid chromatography (HPLC) analysis using an Ultremex fractionation column. The absorbance at 214 nm was monitored and 20 fractions were collected. Peptide pretreatment, procedure optimization, detection of intact peptides in the Orbitrap, and the selection of peptides for MS/MS analysis were carried out using previously described parameters and methods [14] with slight modifications. In this study, a threshold ion count of 20,000 was used in the MS survey scan followed by a 15 s dynamic exclusion duration for precursor ions selection. The electrospray voltage was 1.6 kV, while the automatic gain control (AGC) target was 3e6 for full MS and 1e5 for MS2 to optimize the spectra generated by the Orbitrap. The m/z scan ranges were 350–2000 and 100–1800 Da for MS scans and MS2 scans, respectively.

### 4.3. Peptide and Protein Identification

The raw mass spectrometry data were procedurally converted into the Mascot generic format (MGF) using Proteome Discovery 1.2. The MGF data were then compared against the NCBI non-redundant protein database and the URGI database using the Mascot search engine (Matrix Science, London, UK; version 2.3.02). Because there were more than eight samples, the MGF data were merged into three files during the database search. The Mascot database was set up for protein identification using the fungi-responsive transcriptome of N9134 and the plant species database available at NCBInr/SwissProt/Uniprot/IPI. We used a fragment ion mass tolerance of 0.05 Da and a parent ion tolerance of 10.0 PPM as criteria for the Mascot database search. Identification and quantitation of potential proteins was carried out according to previously described *Bgt* stress conditions [14]. Similarly, differentially accumulated proteins (DAPs) were defined as those with unadjusted *t*-test *p* < 0.05 and ratio of fold changes >1.2 or <0.83. An overall false discovery rate (FDR) was also estimated and the data were analyzed further to identify motifs using InterProScan 5 and the Gibbs Motif Sampler. The identified proteins were annotated with Gene Ontology (GO) terms and Kyoto Encyclopedia of Genes and Genomes (KEGG) pathways using the Molecular Annotation System (http://bioinfo.capitalbio.com/mas). GO functions and KEGG pathways were examined in DAP enrichment analyses. 

### 4.4. Protein–Protein Interaction (PPI) Network Construction

STRING v10.5 [48] was applied to analyze the protein–protein interaction (PPI) of DAPs and DEGs identified in the current study and construct PPI network. The minimum required interaction score parameters was set at the medium confidence level.

### 4.5. Weighted Gene Correlation Network Analysis (WGCNA) and Transcriptome-Proteome-Associated Analysis

To further investigate protein expression profiles following *Pst* CYR31 infection, we carried out transcriptome-proteome-association analysis using the protein and RNA-Seq data. The transcriptome data were obtained from our previous study (NCBI Sequence Read Archive accession number PRJNA243835) [5]. After removal of low quality reads and screening out ribosomal RNA with bowtie [49], all reliable reads were assembled using TopHat2 and Cufflinks [50,51] to reconstruct a gene library referencing the wheat (Chinese spring) genome sequences from URGI (Version v2.2) [52]. Gene expression levels in each sample were evaluated based on the fragments per kilo base of exon model per million of aligned reads (FPKM) values. Differentially expressed genes (DEGs) were selected with DESeq software using the following criteria: fold change ≥2 and FDR ≤0.05. The repeatability of the data for each sample was evaluated by principal component analysis and Pearson correlation analysis of FPKM. Co-expression networks were constructed for transcriptome-proteome-associated analysis using the WGCNA R software package (v1.47) [53] and networks were visualized using Cytoscape_v.3.0.0. Among the 75,906 assembled unigenes, 30,828 genes were selected with FPKM >5 for the WGCNA unsigned co-expression network analysis. Co-expression modules for the large expression database were constructed using the automatic network construction function blockwiseModules with the following settings: power, 10; degree of similarity, 0.75, minModuleSize, 50; mergeCutHeight, 0.3; and TOMType, unsigned. All other parameters of the modules were set to default. The total connectivity, intramodular connectivity (function soft Connectivity), kME (for modular membership, i.e., eigengene-based connectivity), and kME-*p*-values were calculated for all 30,828 genes. Hub genes with potentially important functions were identified as those with high connectivity. 

### 4.6. k-Means Clustering Analysis

Gene expression pattern analysis was used to cluster genes showing similar expression trends at 0, 24, 48, and 72 hpi. To examine the expression pattern of DEGs in each sample, the expression data (in the order of treatment) were normalized to 0, log2 (v1/v0), log2 (v2/v0), log2 (v3/v0), respectively. Clusters were generated using Short Time-series Expression Miner software (STEM, http://www.cs.cmu.edu/~jernst/stem) [54] with the following parameters: (1) Change of maximum unit between time-points, 1; (2) maximum output profiles number, 50 (similar profiles are merged); (3) minimum ratio of fold change of DEGs, ≥2. For the profiles generated, *p* ≤ 0.05 was set as the threshold for statistical significance. All DEGs in each profile were subjected to GO and KEGG pathway enrichment analysis. Following FDR correction, significantly enriched GO terms or pathways were defined as those with *Q*-values ≤ 0.05.

## 5. Conclusions

In this study, we reveal the immense complexity of the mechanisms underlying the responses of wheat to fungal stress. By conducting a comparative quantitative proteomic analysis of the leaves of *Pst*-inoculated wheat variety N9134 at 24, 48 and 72 hpi comparing with mock-inoculated controls, we identified 2050, 2190, and 2258 DAPs, respectively. We then performed a transcriptome-proteome-associated analysis of data from RNA-Seq using WGCNA to predict several key resistance genes. The DEGs identified in this analysis were found to be enriched mainly in four co-expressed gene modules, although the *Pst* infection triggered robust alterations in *T. aestivum* gene expression. A stress stage-specific module was identified exhibiting features of transcription regulator gene enrichment with similar expression patterns. The homologs of several regulators, including splicing and transcription factors, were identified as hub genes operating in the *Pst*-induced response network. Meanwhile, the predicted PPI networks from STRING database substantiated the role of splicing factors as key hub nodes. Additionally, 32 proteins induced in the chromosome 1BS were identified as potential candidates for *Pst*-resistance. Apart from representing a valuable proteomic resource for the characterization of stripe rust resistance pathways at the molecular and biochemical levels, this study provides important insights into the molecular networks underlying the mechanism of wheat defense against *Pst*. The methodology used in this study can also be used as a reference for narrowing down the field of potential key resistance genes using multi-omics and multi-discipline associated analyses, and hence, will be of interest to researchers involved in the study of plant disease-resistant genes.

## Figures and Tables

**Table 1:** Kyoto Encyclopedia of Genes and Genomes (KEGG) pathway enrichment analysis of differentially accumulated proteins in stripe rust infection of wheat variety N9134.

Pathway ID	Pathway Description	DAP Number			*P*/*Q* Value		
		24 hpi	48 hpi	72 hpi	24 hpi	48 hpi	72 hpi
**ko03010**	Ribosome	297 (31.5%)	218 (21.9%)	309 (31.7%)	0*/0	0*/0	0*/0
**ko00190**	Oxidative phosphorylation	124 (13.2%)	192 (19.3%)	112 (11.5%)	0*/0	0*/0	0*/0
**ko00195**	Photosynthesis	133 (14.1%)	95 (9. 6%)	50 (5.1%)	0*/0	0*/0	0.076/0.411
**ko04626**	Plant-pathogen interaction	33 (3.5%)	27 (2.7%)	30 (3.1%)	0*/0	0.002*/0.015	0*/0.002
**ko04145**	Phagosome	26 (2.8%)	26 (2.6%)	20 (2.1%)	0.007*/0.067	0.013*/0.061	0.189/0.758
**ko00260**	Glycine, serine and threonine metabolism	32 (3.4%)	27 (2.7%)	49 (5.0%)	0.001*/0.119	0.041*/0.160	0*/0
**ko00740**	Riboflavin metabolism	–	26 (2.6%)	11 (1.1%)	–	0*/0	0.012*/0.107
**ko01200**	Carbon metabolism	87 (9.2%)	116 (11.7%)	110 (11.3%)	0.085/0.661	0*/0	0*/0.001
**ko00941**	Flavonoid biosynthesis	13 (1.4%)	13 (1.3%)	–	0*/0	0*/0	–
**ko00450**	Selenocompound metabolism	5 (0.5%)	23 (2.3%)	4 (0.4%)	0.721/1	0*/0	0.875/1
**ko00261**	Monobactam biosynthesis	–	22 (2.2%)	–	–	0*/0	–
**ko03022**	Basal transcription factors	–	5 (0.5%)	5 (0.5%)	–	0*/0	0*/0
**ko00020**	Citrate cycle (TCA cycle)	16 (1.7%)	39 (3.9%)	35 (3.6%)	0.684/1	0*/0	0*/0.001
**ko00630**	Glyoxylate and dicarboxylate metabolism	35 (3.7%)	50 (5.0%)	59 (6.0%)	0.193/1	0.001*/0.006	0*/0
**ko00250**	Alanine, aspartate, and glutamate metabolism	13 (1.4%)	22 (2.2%)	19 (1.9%)	0.248/1	0.001*/0.009	0.012/0.107
**ko00920**	Sulfur metabolism	6 (0.6%)	24 (2.4%)	6 (0.6%)	0.989/1	0.003*/0.019	0.992/1
**ko01230**	Biosynthesis of amino acids	48 (5.1%)	82 (8.3%)	76 (7.8%)	0.933/1	0.003*/0.019	0.018/0.143
**ko04712**	Circadian rhythm-plant	15 (1.6%)	13 (1.3%)	–	0*/0.006	0.006*/0.032	–
**ko00480**	Glutathione metabolism	25 (2.6%)	35 (3.5%)	26 (2.7%)	0.24192/1	0.006*/0.032	0.230*/0.826
**ko00670**	One carbon pool by folate	1 (0.1%)	11 (1.1%)	10 (1.0%)	0.997/1	0.031*/0.131	0.061/0.376
**ko00330**	Arginine and proline metabolism	4 (0.4%)	14 (1.4%)	10 (1.0%)	0.956/1	0.032*/0.131	0.278/0.919
**ko00380**	Tryptophan metabolism	6 (0.6%)	–	8 (0.8%)	0.180/1	–	0.041*/0.297

Note: The percentage values shown in brackets are the ratios of differentially accumulated proteins (DAPs) in the corresponding pathway accounting for all annotated differentially accumulated proteins. *p*-values (specific and <0.05) are marked with asterisks. Quality values were shown after the forward slash symbol following the *p*-value.

**Table 2:** The predicted candidate resistance priming gene in wheat variety N9134.

KEGG Pathway	Gene ID	Connectivity	Description
Nuclear envelope	Traes_1BS_7101D8D63	1385.04	SUN domain-containing protein 3-like
Ribosome	Traes_1BS_71C6FDF1A	975.60	Ribosomal protein L28
Ribosome	Traes_1BS_DD122AEA6	41.87	Ribosomal protein S18
Ribosome	Traes_1BS_262C70465	–	Ribosomal protein S19 (chloroplast)
Ribosome	Traes_1BS_F2A8D87C5	–	Ribosomal protein L2 (chloroplast)
Spliceosome	Traes_1BS_68CB9BCB3	23.14	U1 small nuclear ribonucleoprotein A
Spliceosome	Traes_1BS_D86496196	890.18	mRNA transport factor
Protein processing in ER	Traes_1BS_C2166CFB6	726.51	Protein disulfide isomerase-like 2-1
Basal transcription factors	Traes_1BS_30B4D96B4	284.64	Transcription initiation factor TFIID subunit 13
Transcription factors	Traes_1BS_351490964	-	Transcription factor BTF3-like
Plant hormone signal transduction	Traes_1BS_D1FCBFBE8	342.10	Transcription factor PIF3
Plant hormone signal transduction	Traes_1BS_8A19C460B	292.68	Auxin-responsive protein IAA15
Plant hormone signal transduction	Traes_1BS_F1838F858	123.94	Abscisic acid receptor PYL8-like
Arachidonic acid metabolism	Traes_1BS_A6A616FA0	399.75	p23 (Hsp90 co-chaperone)
Insulin resistance	Traes_1BS_BF71914E7	122.99	Ser/thr-protein phosphatase PP1 isozyme 9
Fatty acid elongation	Traes_1BS_F54A0F09C	122.75	3-Ketoacyl-CoA synthase 12
Carotenoid biosynthesis	Traes_1BS_5963FC5FC	109.15	Prolycopene isomerase, chloroplastic-like
Regulation of autophagy	Traes_1BS_C385DE03B	90.79	CBL-interacting protein kinase 17
Flavonoid biosynthesis.	Traes_1BS_B00164D10	47.44	Chalcone-flavonone isomerase 1B-1
Defense	Traes_1BS_2BCFB8A20	182.20	Subtilisin-chymotrypsin inhibitor-2A
Selenocompound metabolism	Traes_1BS_B67548763	35.60	Methionine S-methyltransferase
Val, leu and isoleucine degradation	Traes_1BS_190C56A4E	30.76	Dihydrolipoyl dehydrogenase 1, mitochondrial
Peroxisome	Traes_1BS_A344E8CC2	24.82	Peroxisomal membrane protein 2
Peroxisome	Traes_1BS_40116F0E7	10.18	Peroxisomal membrane protein PEX14-like
Glutathione metabolism	Traes_1BS_F226DF9B4	368.85	Dehydroascorbate reductase
Oxidative phosphorylation	Traes_1BS_6F8E6B9B6	–	ATP synthase protein atp8-1(3 copes in 1BS)
Cell cycle	Traes_1BS_4E2FA3101	–	TPA: histone H4.3
Fructose and mannose metabolism	Traes_1BS_0039045F7	215.75	Fructose-2, 6-biphosphatase 1
Pyruvate metabolism	Traes_1BS_478039FB4	201.84	Probable lactoylglutathione lyase, chloroplast
Porphyrin metabolism	Traes_1BS_ABD495014	154.21	Glutamyl tRNA reductase

## Supplementary Materials

Supplementary materials can be found at https://www.mdpi.com/1422-0067/20/5/1106/s1. The proteome data from this study have been submitted to http://www.peptideatlas.org/ under accession Nos. PeptideAtlas PASS00999. The following materials are available in the online version of this article: Figure S1. Gene ontology classifications of the DAPs in *Pst*-infected wheat leaves at 48 and 72 hpi. Figure S2. Clustering of the pathogen induced-specific genes module eigengene. Figure S3. *Pst*-induced-specific genes in mediumpurple2 and lightpink4 module and their enrichment pathways. Figure S4. A. Clustering of the patterns of DE genes. B. Bubble chart of the top 20 enriched pathways in profiles 7 and 18. Table S1. Gene ontology function enrichment analysis of DAPs involved in Pst-stress PPI network construction. Table S2. Significant KEGG enrichment pathway of genes involved in specific modules. Table S3. The main hub genes with high connectivity involved in *Pst* stress-specific modules. Table S4. The top KEGG pathways with high representation of the DEGs in specific profiles. Table S5. Function enrichment analysis of disease resistance related proteins and transcript DEGs involved in Pst-stress PPI network construction. Table S6. iTRAQ tag types and concentration of the samples.
