# Supplementary material for: Transcriptome and Proteome-Based Network Analysis Reveals a Model of Gene Activation in Wheat Resistance to Stripe Rust
Source: Int J Mol Sci. 2019 Mar 4;20(5):1106. doi: 10.3390/ijms20051106 (PMC6429138; doi:10.3390/ijms20051106)
Supplement: Supplementary file 1 [file ijms-20-01106-s001.pdf]

Table S1. Gene ontology function enrichment analysis of DAPs involved in Pst-stress PPI network construction.

STRING Protein	STRING Protein Description	STRING Protein	STRING Protein Description
LEA14	Late embryogenesis abundant 14	AT3G15220	Putative protein kinase
SAM1	S-adenosylmethionine synthetase 1	CTL2	Chitinase-like protein 2
SPS3F	Sucrose-phosphate synthase	AT3G18190	TCP-1/cpn60 chaperonin family protein
PDIL2-2	PDI-like 2-2(protein disulfide isomerase)	AT3G28710	V-type proton ATPase subunit d1
AT1G05240	Peroxidase 1/2	HIS4	Histone H4
RCI3	Peroxidase 3	PHS2	Alpha-glucan phosphorylase 2
CRT3	Calreticulin 3	CHL	Chloroplastic lipocalin
PDF1	Defensin-like protein	ATPQ	ATP synthase subunit d
UBC36	Ubiquitin-conjugating enzyme 36	SBPASE	Sedoheptulose-bisphosphatase
CLH1	Chlorophyllase 1	BGL2	Beta 1,3-glucanase
AT1G23140	Calcium-dependent lipid-binding domain-containing protein	CYSC1	Cysteine synthase C1
AT4G03120	C2H2 and C2HC containing protein (Component of the U1 snRNP)	AT1G24110	Peroxidase 6
AT1G30870	Peroxidase 7	CYS2	Cysteine proteinase inhibitor 2
ACBP6	Acyl-CoA-binding protein 6	AT4G17070	Peptidyl-prolyl cis-trans isomerase
SERPIN1	Serpin-ZX	SHM3	Serine hydroxymethyltransferase 3
NHO1	Glycerol kinase	AT4G33420	Peroxidase
PGR5	Proton gradient regulation 5	CAT	Catalase 2
AGT	Alanine-glyoxylate aminotransferase	PRX52	Peroxidase 52
PR1	Pathogenesis-related gene 1	PA2	Peroxidase 2
TIM	Triosephosphate isomerase	AT5G06730	Peroxidase 54
PAG1	20S proteasome alpha subunit G1	PMDH2	Malate dehydrogenase
FBA6	Aldolase superfamily protein	ATP5	ATP synthase subunit O
OASB	Cysteine synthase	AT5G14030	Translocon-associated protein subunit beta
U1A	Spliceosomal protein U1A	AT5G20090	Uncharacterized protein
AT5G18140	DnaJ domain-containing protein	AT5G47030	ATP synthase subunit delta’
MDHAR	Monodehydroascorbate reductase	SGT1	Suppressor of the G2 allele of skp1
AT3G11830	TCP-1/cpn60 chaperonin family protein	AT5G51890	Peroxidase 66
HSP70	Heat shock protein 70	STR16	Sulfurtransferase protein 16

Table S2. Significant KEGG enrichment pathway of genes involved in specific modules.

Pathway ID	Pathway	correction P value				
		lightpink4	mediumpurple2	coral2	skyblue2	lightcyan
**ko01200**	Carbon metabolism	0	0.008844			0.00218
**ko03040**	Spliceosome				0	0.000479
**ko00630**	Glyoxylate and dicarboxylate metabolism	0				0.026698
**ko04075**	Plant hormone signal transduction		0			
**ko00620**	Pyruvate metabolism	0.000531	0.008116	0.020014		
**ko00260**	Glycine, serine and threonine metabolism	0	0.011032			
**ko00196**	Photosynthesis - antenna proteins		0	0		0.000006
**ko00860**	Porphyrin and chlorophyll metabolism		0.000002	0.000035		
**ko00010**	Glycolysis / Gluconeogenesis	0.001529				
**ko00020**	Citrate cycle (TCA cycle)	0.011216				
**ko00030**	Pentose phosphate pathway	0.006464				
**ko00051**	Fructose and mannose metabolism	0.004619				
**ko00052**	Galactose metabolism		0.000288			
**ko00053**	Ascorbate and aldarate metabolism			0.039394	0.013897	
**ko00061**	Fatty acid biosynthesis	0.001694				
**ko00130**	Ubiquinone and other terpenoid-quinone biosynthesis	0.043665	0.011539			
**ko00190**	Oxidative phosphorylation			0.047802		
**ko00195**	Photosynthesis					0.000346
**ko00220**	Arginine biosynthesis	0.026941				
**ko00261**	Monobactam biosynthesis	0.019113				
**ko00270**	Cysteine and methionine metabolism		0.000055		0.007812	
**ko00330**	Arginine and proline metabolism		0.032281		0.006261	
**ko00350**	Tyrosine metabolism		0.016708			
**ko00410**	beta-Alanine metabolism				0.002066	
**ko00450**	Selenocompound metabolism	0.042452				
**ko00460**	Cyanoamino acid metabolism	0.002224				
**ko00480**	Glutathione metabolism				0.000119	0.016627
**ko00500**	Starch and sucrose metabolism	0.001873	0.041555			
**ko00561**	Glycerolipid metabolism	0.024711	0.000098			
**ko00562**	Inositol phosphate metabolism		0.033962			
**ko00650**	Butanoate metabolism			0.035547		
**ko00660**	C5-Branched dibasic acid metabolism	0.045943				
**ko00670**	One carbon pool by folate	0.008167				
**ko00710**	Carbon fixation in photosynthetic organisms	0.000045				
**ko00740**	Riboflavin metabolism	0.000006				0.024943
**ko00760**	Nicotinate and nicotinamide metabolism			0.018258		
**ko00770**	Pantothenate and CoA biosynthesis	0.042452				
**ko00780**	Biotin metabolism	0.014544				
**ko00790**	Folate biosynthesis	0.028386				
**ko00903**	Limonene and pinene degradation			0.001218	0.014485	
**ko00906**	Carotenoid biosynthesis	0.014422				
**ko00910**	Nitrogen metabolism	0.048367				
**ko00920**	Sulfur metabolism	0.016296				
**ko00945**	Stilbenoid, diarylheptanoid and gingerol biosynthesis				0.048814	
**ko00950**	Isoquinoline alkaloid biosynthesis		0.016726			
**ko00960**	Tropane, piperidine and pyridine alkaloid biosynthesis		0.001566			
**ko00970**	Aminoacyl-tRNA biosynthesis	0.001063				
**ko01210**	2-Oxocarboxylic acid metabolism	0.002649				
**ko01212**	Fatty acid metabolism	0.04141				
**ko01230**	Biosynthesis of amino acids	0				
**ko03010**	Ribosome			0.000001		
**ko03050**	Proteasome					0.008979
**ko03060**	Protein export	0.000185				
**ko04145**	Phagosome					0.007882
**ko04146**	Peroxisome	0.034139				0.047956
**ko04712**	Circadian rhythm - plant		0.009379			
**ko04931**	Insulin resistance	0.036556				

Table S3. The main hub genes with high connectivity involved in Pst stress-specific modules.

GeneID	Connectivity	Description	Species
**Traes_2AL_158F8EE88**	516.686479	pathogen-inducible transcription factor ERF3	Ta
**Traes_5DL_502C1AC95**	274.9678487	CBL-interacting protein kinase 14	Hv
**Traes_1BS_CEBFF8BEB**	251.7319399	secologanin synthase-like	Bd
**Traes_5BL_5897DA404**	248.7938523	Transcription elongation factor A protein 2	Tu
**Traes_1BS_F54A0F09C**	239.4543802	3-ketoacyl-CoA synthase 12	At
**Traes_1DL_A530B3014**	232.5886235	Splicing factor U2af small subunit B	At
**Traes_5BL_B7BBD43E1**	232.4885047	pre-mRNA processing factor	Ta
**Traes_6BS_A462CA491**	230.7806005	Serine/threonine-protein kinase HT1	Tu
**Traes_5DL_3DC0ED961**	228.112008	mediator of RNA polymerase II transcription subunit 14-like	Si
**Traes_3B_5CABEBCE5**	220.0607436	transcription factor HBP-1b(c38)-like	Bd
**Traes_3B_D8B3F965A**	219.899133	coronatine insensitive 2-like protein	Ta
**Traes_1AS_5F6AE0420**	203.3766292	GSK-like kinase	Ta
**Traes_3B_3D1F3FBC5**	200.1094452	Neurofilament heavy polypeptide	Tu
**Traes_4AL_2BCB74D01**	199.5302198	DEAD-box ATP-dependent RNA helicase 21	Tu
**Traes_4AS_6D7CDA716**	198.4536263	Serine/threonine-protein phosphatase PP1	At
**Traes_1DS_4BB707532**	188.6883642	abscisic acid receptor PYL8-like	Bd
**Traes_7DL_21CCF6E42**	187.991055	pre-mRNA-splicing factor syf2-like	Bd
**Traes_6AL_9FD263FF1**	181.6420073	mRNA decapping complex subunit 2	At
**Traes_1BS_C385DE03B**	178.0844507	CBL-interacting protein kinase 17	Hv
**Traes_4DL_FFD4866F3**	174.2871091	Heat shock cognate 70 kDa protein	At
**Traes_6DL_6DC301F80**	173.0007335	Serine/arginine-rich splicing factor 7	Tu
**Traes_4DS_5AAF38D73**	172.0194585	Serine/threonine-protein kinase CTR1	Tu
**Traes_1DL_E329E0435**	170.758998	coronatine insensitive 1-like protein	Ta
**Traes_6BS_E699D8578**	163.9342056	Signal recognition particle receptor subunit alpha	At
**Traes_1BS_BF71914E7**	160.6819652	Serine/threonine-protein phosphatase PP1 isozyme 9	At
**Traes_7DS_8C8A5B578**	160.1176271	CRM domain-containing protein At3g25440, chloroplastic-like	Bd
**Traes_2BS_7FA2FCB85**	154.2647467	pre-mRNA-splicing factor SYF1-like	Bd
**Traes_5BL_6CFCBCCE6**	153.8157078	Transcription factor PIF5	At
**Traes_7DL_F451957CD**	152.521758	serine/threonine protein kinase 1	Ta
**Traes_4BL_4D370C9FA**	145.8287231	CytHSP70	Ta
**Traes_2AL_73E4240F0**	141.6273024	Trihelix transcription factor GT-1	Tu
**Traes_3AS_DD21AD44C**	139.1983977	Eukaryotic translation initiation factor 6-2	Tu
**Traes_3AL_E77F7C3EE**	135.3849364	Auxin-responsive protein IAA6	Tu
**Traes_2DS_468F40660**	126.5324017	DNA-directed RNA polymerases I, II, and III subunit RPABC2-like	Bd
**Traes_1BS_BEC0ABF3A**	121.5361813	E3 ubiquitin-protein ligase XB3-like	Bd
**Traes_1AS_7CB2CE2FF**	119.4866867	probable calcium-binding protein CML18-like	Bd
**Traes_2AL_C37FA7FDC**	118.7321629	Serine/threonine-protein kinase SAPK5	Tu
**Traes_6DS_0A9DA46CE**	117.5917541	pollen-specific protein SF21-like	Bd
**Traes_1BS_29834F374**	117.2086178	Tuftelin-interacting protein 11	At
**Traes_6AL_5B4E3D226**	115.9904896	pathogen induced protein 2-4	Hv
**Traes_2DS_62C233677**	115.7480641	60S ribosomal protein L18-2	At
**Traes_1BS_B734EDEA7**	115.2057593	zinc transporter 6-like	Bd
**Traes_7DL_7AF52054F**	112.7066	Glycine-rich RNA-binding protein 2	Tu
**Traes_4BS_64C1860C6**	111.3225947	Transcriptional corepressor SEUSS	Tu
**Traes_5DL_9BFC56771**	88.52969321	WD repeat-containing protein 20-like	Bd
**Traes_5BL_C52B6F6D6**	87.35219578	rust resistance gene ABC1041	Hv
**Traes_7DL_D58ABAE70**	86.67951527	ko03040//Spliceosome	-
**Traes_6DS_8F56F52ED**	82.65930504	probable WRKY transcription factor 19-like isoform X1	Si

**Traes_1BS_68CB9BCB3**	80.28277204	U1 small nuclear ribonucleoprotein A	At
**Traes_7AS_F4CF6E7E0**	79.7585947	Calcium-dependent protein kinase 3	Tu
**Traes_5AL_81602ECE9**	70.12111526	multidrug resistance-associated protein MRP1, partial	Ta
**Traes_3AS_8107B5E92**	68.64413491	60S ribosomal protein L26-1	Tu
**Traes_5BL_F08C5D7AE**	66.88570774	Glycine-rich RNA-binding protein 2, mitochondrial	At
**Traes_4BL_03EF0DD06**	65.96518485	pre-mRNA-splicing factor cwc-25-like	Si
**Traes_1BS_3761C242C**	59.10600376	GATA transcription factor 22	At
**Traes_7BS_32C1D0852**	48.24774262	uncharacterized WD repeat-containing protein C2A9.03-like	Bd
**Traes_5AL_9B10F93AC**	34.84439057	THO complex subunit 4-A	At
**Traes_7AL_354EEE44E**	30.18964794	Os06g0335500	Os
**Traes_5DL_F2955A6D3**	27.88388274	Ras-related protein Rab11A	At
**Traes_1DL_3FB439B4E**	27.23455133	RNA-binding Musashi-2-like protein	At
**Traes_7DL_0FFDD50EE**	26.58383208	U6 snRNA-associated Sm-like protein LSm7-like	Bd
**Traes_2AL_66B5C77CB**	25.11805838	small nuclear ribonucleoprotein-associated protein B'-like	Sl
**Traes_2DS_9DC4D07BB**	23.80077502	serine/arginine repetitive matrix protein 1-like isoform X1	Si
**Traes_3DS_0EC06EE9D**	12.05884831	DEAD-box ATP-dependent RNA helicase 40	At
**Traes_1BS_C7F30A549**	8.571439111	Na+/H+ antiporter NHX1	Le
**Traes_4DL_08B1BCAF6**	8.115780725	WD repeat-containing protein 44	Tu

Table S4. The top KEGG pathways with high representation of the DEGs in specific profiles.

Pathway	Pathway ID	All_profiles (3488)	Profile7 (1053)	Profile8 (321)	Profile9 (167)	Profile10 (298)	Profile15 (165)	Profile16 (83)	Profile17 (252)	Profile18 (315)
**Plant hormone signal transduction**	ko04075	150(4.30%)	27(2.56%)	2(0.62%)	12(7.19%)	24(8.05%)	2(1.21%)	1(1.20%)	16(6.35%)	33(10.48%)
**Plant-pathogen interaction**	ko04626	115(3.30%)	25(2.37%)	12(3.74%)	10(5.99%)	10(3.36%)	6(3.64%)	1(1.20%)	10(3.97%)	19(6.03%)
**Ribosome biogenesis in eukaryotes**	ko03008	104(2.98%)	58(5.51%)	16(4.98%)	0(0.00%)	7(2.35%)	2(1.21%)	2(2.41%)	1(0.40%)	3(0.95%)
**Spliceosome**	ko03040	102(2.92%)	27(2.56%)	9(2.80%)	0(0.00%)	7(2.35%)	4(2.42%)	1(1.20%)	6(2.38%)	20(6.35%)
**Insulin resistance**	ko04931	69(1.98%)	16(1.52%)	6(1.92%)	3(1.80%)	11(3.69%)	3(1.82%)	0(0.00%)	4(1.59%)	8(2.54%)
**Photosynthesis - antenna proteins**	ko00196	62(1.78%)	1(0.09%)	1(0.31%)	20(11.98%)	4(1.34%)	0(0.00%)	0(0.00%)	20(7.94%)	2(0.63%)
**Glycerolipid metabolism**	ko00561	57(1.63%)	3(0.28%)	4(1.25%)	3(1.80%)	14(4.70%)	5(3.03%)	0(0.00%)	7(2.78%)	14(4.44%)
**Phenylalanine metabolism**	ko00360	57(1.63%)	18(1.71%)	2(0.62%)	4(2.40%)	3(1.01%)	0(0.00%)	0(0.00%)	7(2.78%)	12(3.81%)
**Phagosome**	ko04145	52(1.49%)	10(0.95%)	7(2.18%)	3(1.80%)	2(0.67%)	9(5.45%)	5(6.02%)	1(0.40%)	3(0.95%)
**Regulation of autophagy**	ko04140	44(1.26%)	5(0.47%)	2(0.62%)	5(2.99%)	9(3.02%)	2(1.21%)	0(0.00%)	4(1.59%)	6(1.90%)
**Ascorbate and aldarate metabolism**	ko00053	42(1.20%)	22(2.09%)	3(0.93%)	1(0.60%)	5(1.68%)	1(0.61%)	0(0.00%)	1(0.40%)	1(0.32%)
**Carotenoid biosynthesis**	ko00906	34(0.97%)	21(1.99%)	3(0.93%)	0(0.00%)	1(0.34%)	1(0.61%)	0(0.00%)	0(0.00%)	1(0.32%)
**beta-Alanine metabolism**	ko00410	34(0.97%)	3(0.28%)	3(0.93%)	4(2.40%)	5(1.68%)	0(0.00%)	0(0.00%)	5(1.98%)	5(1.59%)
**Monobactam biosynthesis**	ko00261	18(0.52%)	9(0.85%)	4(1.25%)	0(0.00%)	0(0.00%)	0(0.00%)	1(1.20%)	0(0.00%)	0(0.00%)

Table S5. Function enrichment analysis of disease resistance related proteins and transcript DEGs involved in Pst-stress PPI network construction.

STRING Protein	STRING Protein Description	STRING Protein	STRING Protein Description
**TAF13**	TBP-associated factor 13	**AT3G06480**	DEAD box RNA helicase family protein
**CRTISO**	Carotenoid isomerase	**MDHAR**	Monodehydroascorbate reductase
**AT1G07210**	Ribosomal protein S18	**HSP70**	Heat shock protein 70
**PIF3**	Transcription factor PIF3	**LPD1**	Dihydrolipoyl dehydrogenase 1
**RPS5**	RESISTANT TO P. SYRINGAE 5	**TDX**	TPR repeat-containing thioredoxin TDX
**GT-1**	GT-1(Trihelix transcription factor)	**LOH1**	Uncharacterized CRM domain-containing protein
**BTF3**	Basic transcription factor 3	**RABA2c**	RAB GTPase homolog A2C
**CDPK1**	Calcium-dependent protein kinase 10	**AT3G49910**	60S ribosomal protein L26-1
**DHAR1**	Dehydroascorbate reductase	**PDIL1-3**	PDI-like 1-3
**AT1G22882**	SUN domain-containing protein	**eIF6A**	Eukaryotic initiation factor 6A
**ABCC1**	ATP-binding cassette C1	**BGL2**	Beta 1,3-glucanase
**SEU**	SEUSS transcriptional co-regulator	**PIL6**	Transcription factor PIF5
**CIPK17**	CBL-interacting protein kinase 17	**MEKK1**	MAPK/ERK kinase kinase 1
**AT1G51380**	DEAD-box ATP-dependent RNA helicase 34	**WRKY19**	Putative WRKY transcription factor 19
**IAA6**	Indole-3-acetic acid 6	**GR-RBP2**	Glycine-rich RNA-binding protein 2
**HT1**	High leaf temperature 1	**SGT1**	Suppressor of the G2 allele of skp1
**SS13**	Strictosidine synthase 3	**CDPK6**	Calcium-dependent protein kinase 6
**IAA15**	Auxin-responsive protein IAA15	**AT4G25550**	Cleavage/polyadenylation specificity factor
**NHO1**	Glycerol kinase	**CGA1**	Cytokinin-responsive gata factor 1
**EMB2816**	EMBRYO DEFECTIVE 2816	**AT4G30600**	Signal recognition particle receptor subunit alpha
**PR1**	Pathogenesis-related gene 1	**AKR2**	Ankyrin repeat-containing protein 2
**AT2G16860**	pre-mRNA splicing factor SYF2	**CIPK14**	Serine/threonine protein kinase 1
**KCS12**	3-ketoacyl-CoA synthase 12	**CTR1**	CONSTITUTIVE TRIPLE RESPONSE 1
**TOPP1**	Type one protein phosphatase 1	**CPK1**	Calcium-dependent protein kinase 1
**ZIP6**	Zinc transporter 6	**DCP2**	mRNA-decapping enzyme subunit 2
**HEMA3**	glutamyl-tRNA reductase	**U2AF35B**	Splicing factor U2af small subunit B
**PRPL28**	60S ribosomal protein L28-1	**PDF1.2**	Defensin-like protein 268
**AT2G33730**	DEAD-box ATP-dependent RNA helicase 21	**At5g47320**	Ribosomal protein S19
**AT2G38390**	Peroxidase 23	**MMT**	Methionine S-methyltransferase
**COI1**	CORONATINE INSENSITIVE 1	**ILR3**	bHLH transcription factor ILR3
**U1A**	Spliceosomal protein U1A	**TEX1**	THO complex subunit 3
**AT3G01520**	Universal stress protein A-like protein	**AT5G59950**	THO complex subunit 4
**AT3G03000**	Putative calcium-binding protein CML18	**PEX14**	Peroxin 14
**SWP**	Mediator of RNA polymerase II transcription subunit 14	**RPSL2**	Ribosomal protein L2
**RPL18**	Ribosomal protein L18		

Table S6. iTRAQ tag types and concentration of the samples.

Sample	iTRAQ tag	Concentration(μg/μl)
9134_0h_R1	114	6.09
9134_0h_R2	114	12.03
9134_0h_R3	114	21.9
9134_24h_R1	115	17.68
9134_24h_R2	115	20.12
9134_24h_R3	115	23.21
9134_48h_R1	116	11.87
9134_48h_R2	116	10.28
9134_48h_R3	116	16.78
9134_72h_R1	118	17.68
9134_72h_R2	118	17.43
9134_72h_R3	118	17.34

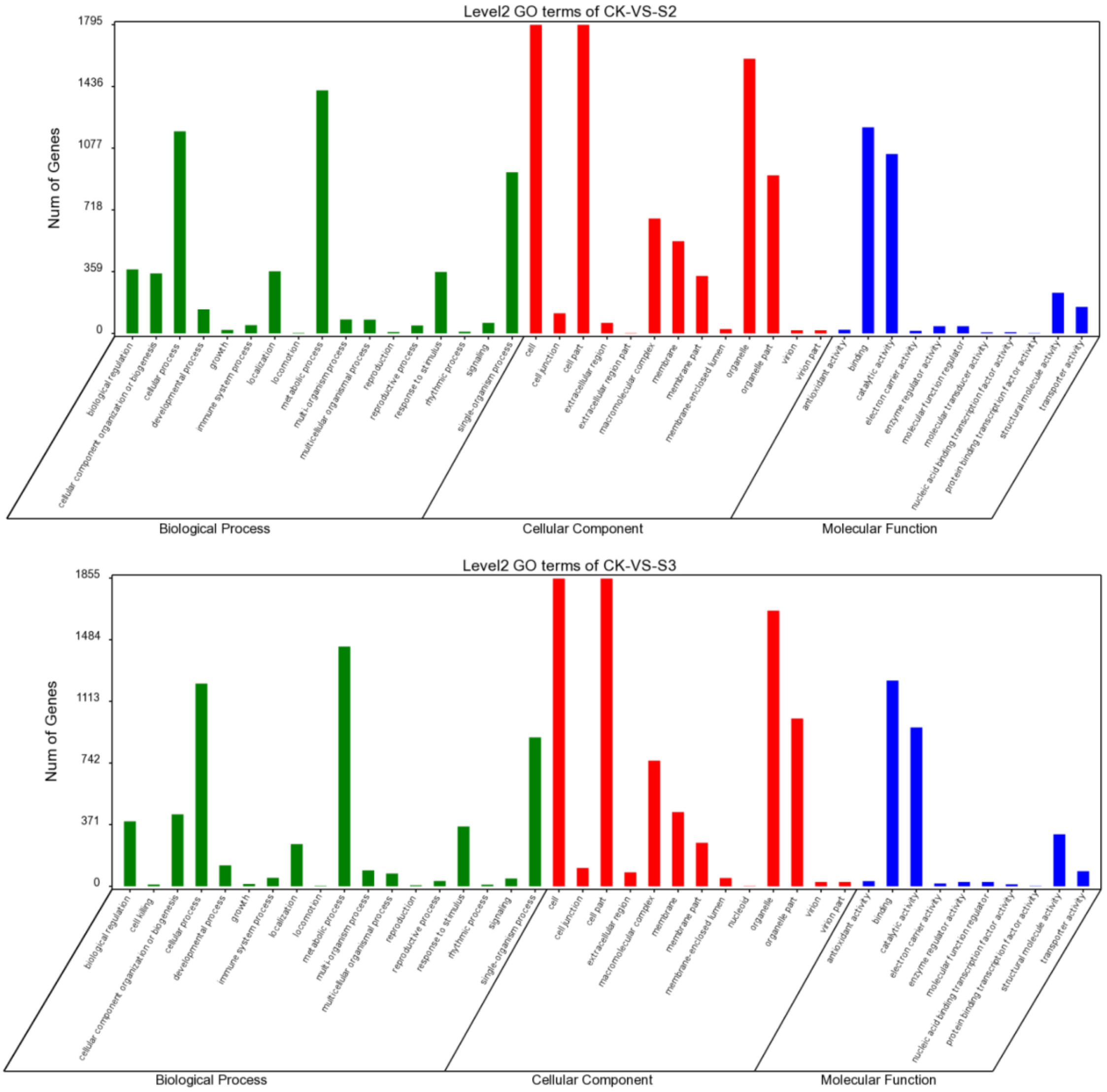

Figure S1. Gene ontology classifications of the DAPs in *Pst*-infected wheat leaves at 48 (Ck-Vs-S2) and 72 hpi (Ck-Vs-S3).

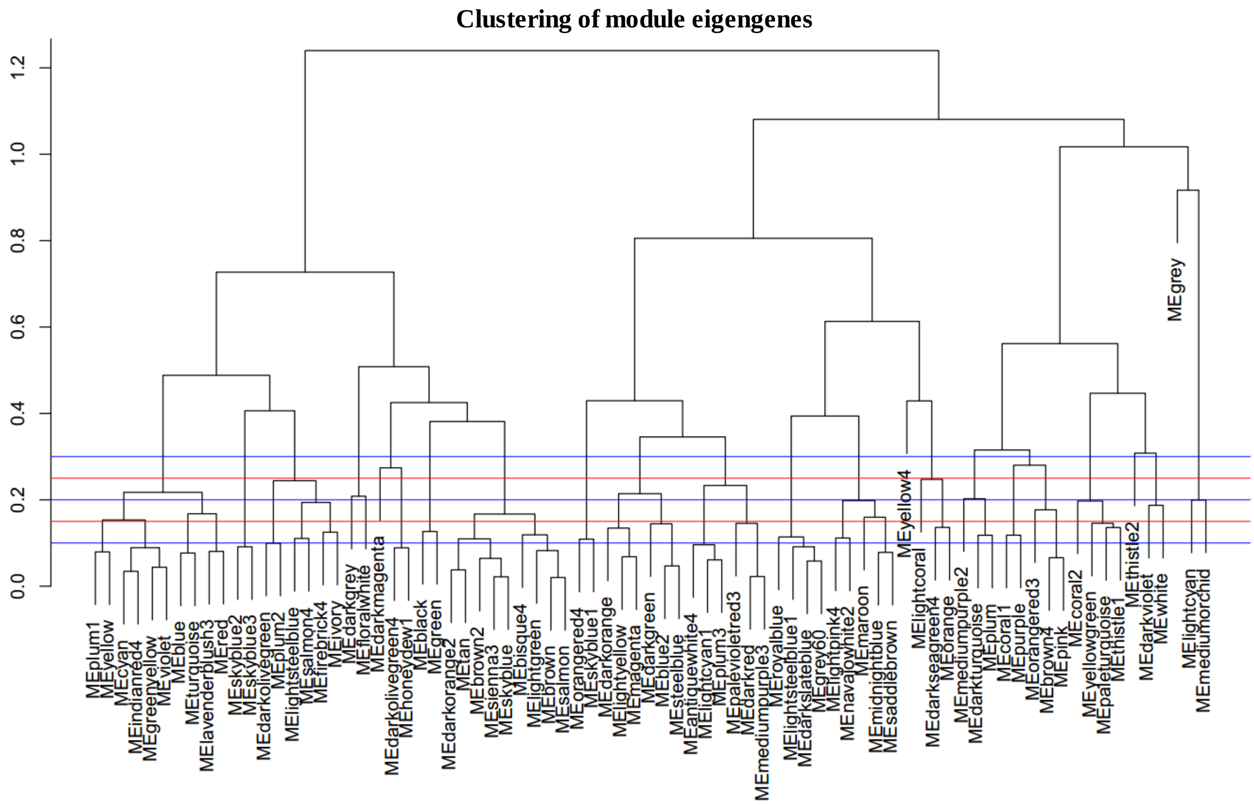

Figure S2. Clustering of the pathogen induced-specific genes module eigengene. The scale was given in the left and indicated the value of the mergeCutHeight.

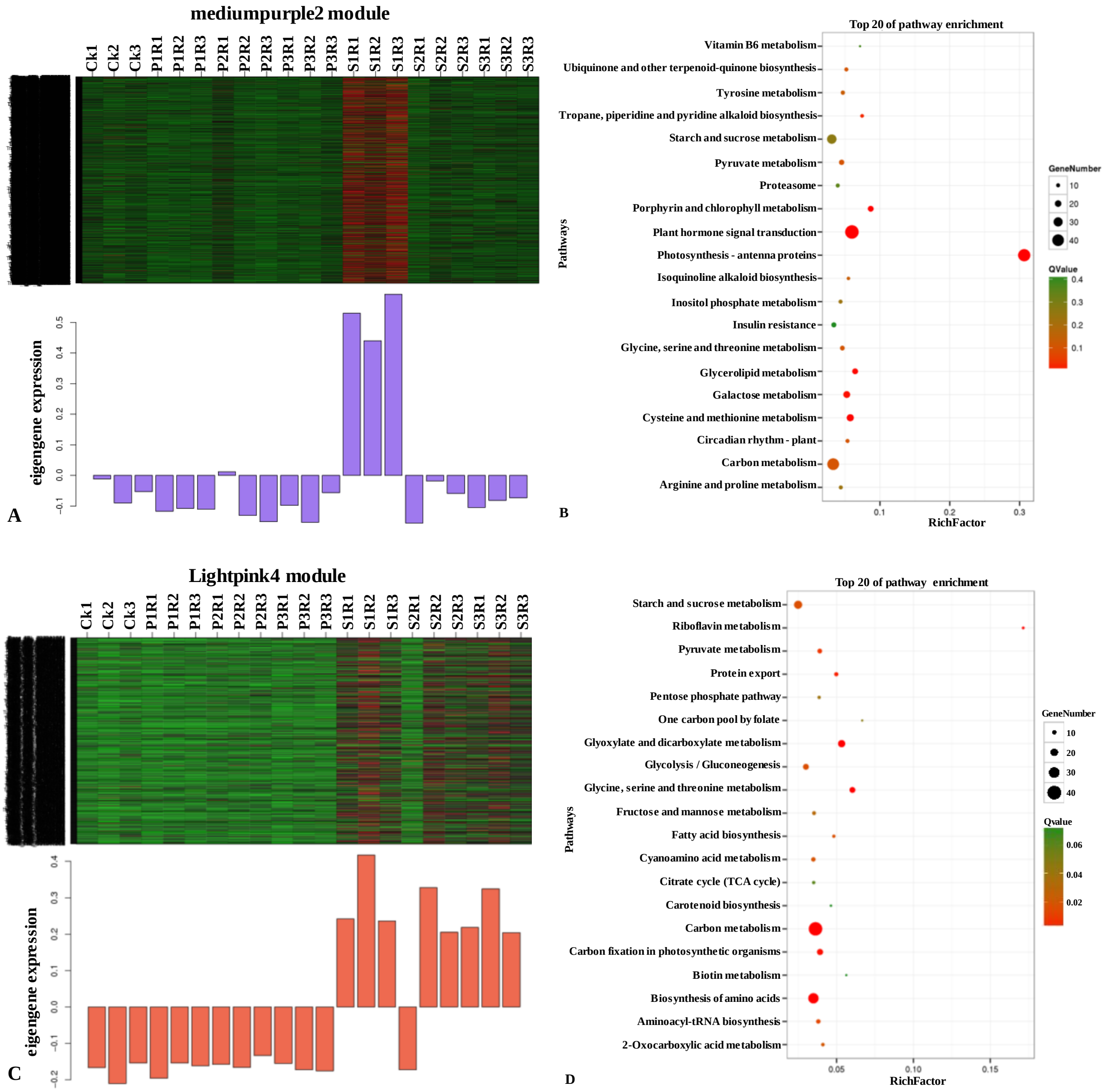

Figure S3. *Pst*-induced-specific genes in mediumpurple2 and lightpink4 module and their enrichment pathways. A and C, the hot maps and Eigengene expression profile for the mediumpurple 2 and lightpink4 module respectively in inoculated leaves of N9134. The y axis indicates the value of the module eigengene; the x axis indicates sample type. Heat map showing the relative NRPKM of each gene. B and D, the top 20 of KEGG enrichment of DEGs in each corresponding module.

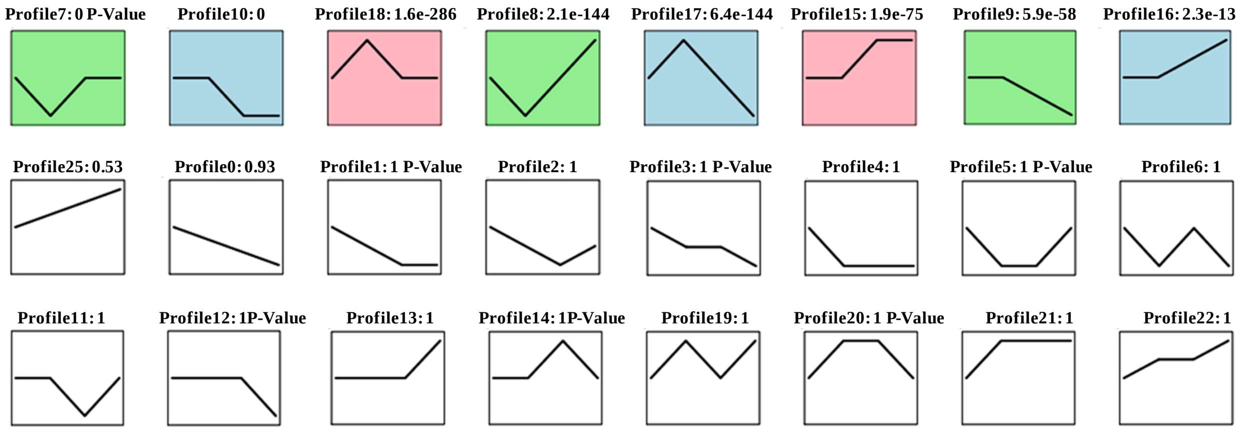

Figure S4A. Clustering of the patterns of DE genes. The expression patterns of 12727 DE genes for 24 clusters. The horizontal axis indicates each time point (0, 1, 2 and 3 dpi) under *Pst* CYR 31 pathogen stress induction condition. The vertical axis indicates the log2 fold change calculated between each condition and non-inoculation treatment. Each bold back line illustrates the average expression pattern of all genes in each cluster.

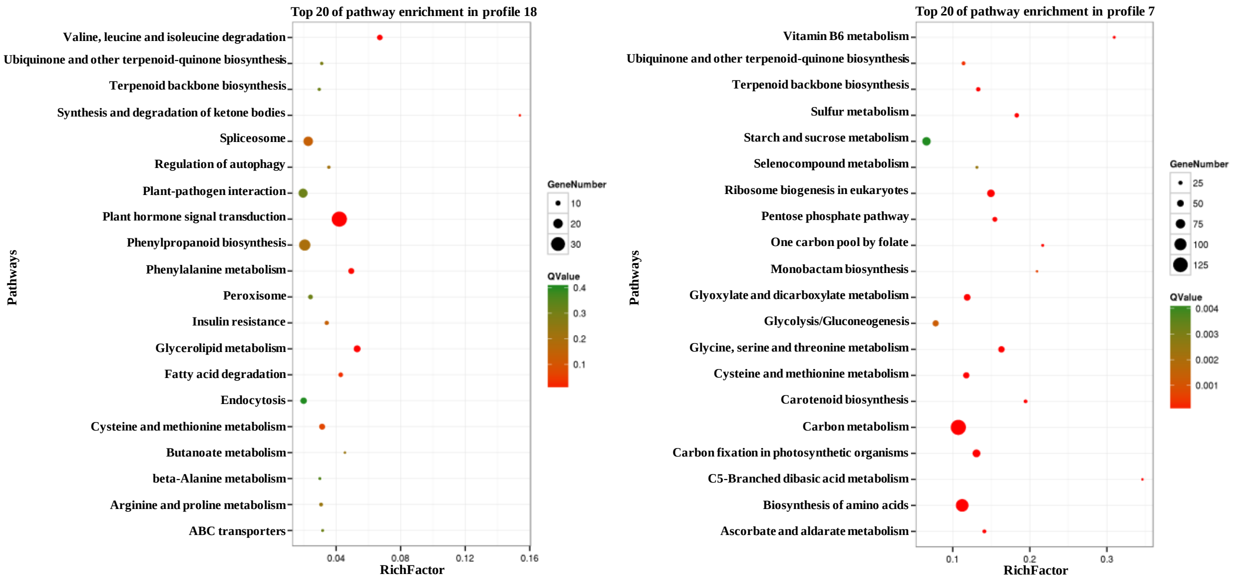

Figure S4B. Bubble chart of the top 20 enriched pathways in profiles 7 and 18.
